# Vocal cord paralysis in Charcot–Marie–Tooth type 4b1 disease associated with a novel mutation in the myotubularin-related protein 2 gene: A case report and review of the literature

**DOI:** 10.1016/j.nmd.2017.01.006

**Published:** 2017-05

**Authors:** Alberto Andrea Zambon, Maria Grazia Natali Sora, Giovanna Cantarella, Federica Cerri, Angelo Quattrini, Giancarlo Comi, Stefano Carlo Previtali, Alessandra Bolino

**Affiliations:** a Department of Neurology, San Raffaele Scientific Institute, Via Olgettina 60, 20132 Milan, Italy; b Otolaryngology Department, Fondazione IRCCS Ca' Granda Ospedale Maggiore Policlinico, Via F. Sforza 35, Milan, Italy; c Experimental Neuropathology Unit, INSPE and Division of Neuroscience, San Raffaele Scientific Institute, Via Olgettina 60, 20132 Milan, Italy; d Neuromuscular Repair Unit, INSPE and Division of Neuroscience, San Raffaele Scientific Institute, Via Olgettina 60, 20132 Milan, Italy; e Human Inherited Neuropathies Unit, INSPE and Division of Neuroscience, San Raffaele Scientific Institute, Via Olgettina 60, 20132 Milan, Italy

**Keywords:** CMT4B1, MTMR2, Vocal cord palsy, Autosomal recessive neuropathy, Vocal fold laterofixation

## Abstract

Charcot–Marie–Tooth type 4B1 (CMT4B1) is an autosomal recessive motor and sensory demyelinating neuropathy characterized by the association of early-onset neurological symptoms and typical histological findings. The natural history and the clinical variability of the disease are still poorly known, thus further clarification of the different phenotypes is needed. We report on the case of a Pakistani girl born to consanguineous parents harboring a novel mutation in the *MTMR2* gene. When aged 18 months, reduced limb tone, muscle wasting associated with proximal and distal weakness prevalent in lower limbs, absence of tendon reflexes, hoarseness and inspiratory stridor were detected. Vocal cord palsy was diagnosed shortly after. We suggest that laryngeal involvement might be a relevant and initial feature of early-onset CMT4B1 neuropathy. Thus, affected patients should undergo early laryngological evaluation in order to prompt an appropriate management.

## Highlights

• Vocal cord paralysis is a relevant symptom of Charcot–Marie–Tooth type 4B1.
• Patients harboring *MTMR2* mutations should be investigated for laryngeal function.
• A new mutation in the MTMR2 gene is described.
• The frequency of vocal cord paralysis in early-onset CMT subtypes is explored.

## 1 Introduction

Charcot–Marie–Tooth neuropathy (CMT) represents a widely heterogeneous group of disorders with a broad spectrum of clinical and electrophysiological features. More than 80 genes have been identified so far as responsible for CMT, including dominant, recessive and X-linked forms [1].

CMT type 4B1 (CMT4B1) is an autosomal recessive (AR) motor and sensory demyelinating neuropathy characterized by the association of early-onset neurological symptoms and typical biopsy findings. The histopathological hallmark is the presence of myelin outfoldings in the nerve, redundant and irregular loops of myelin around the axon [2]. The disease is caused by loss-of-function mutations in the Myotubularin-related 2 gene (*MTMR2*) on chromosome 11q22 [3].

To date, few cases have been reported and further clarification of the different phenotypes associated with this gene is needed, particularly in consideration of the poor knowledge on the natural history of the disease and its severe course, which may need to provide clinical warnings to care-givers.

We here report the case of a patient with a novel mutation in the *MTMR2* gene, who started to experience stridor and was diagnosed with bilateral vocal cord paralysis at the age of 18 months.

## 2 Case report

### 2.1 Clinical and electrophysiological phenotype

**Fig. 1:**
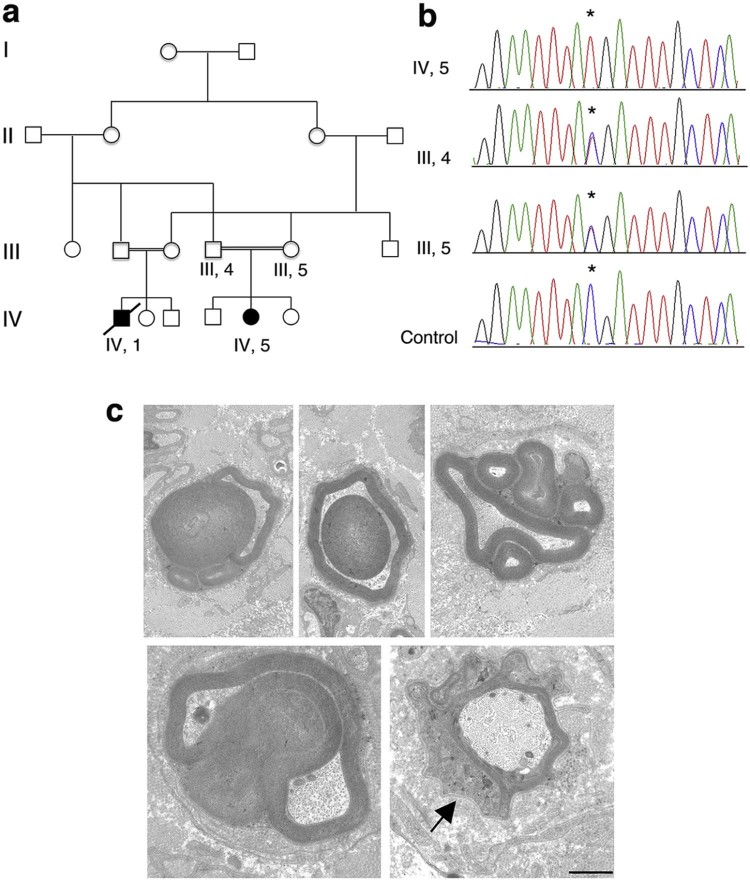
Molecular analysis and nerve biopsy. (a) Pedigree of the reported family with two affected individuals, first cousins. (b) Electropherograms of exon 6 of the *MTMR2* gene show that the c.484 C>T (p.Arg162*) mutation is present in heterozygosity in the unaffected parents and in homozygosity in the proband (IV, 5). The asterisk in the electropherograms indicates the mutation. (c) Sural nerve biopsy of the proband revealed the presence of fibers with outfoldings, infoldings and with redundant basal lamina (arrow). Bar is 1 µm.

The proband is a Pakistani girl born to consanguineous parents (first cousins) after uneventful pregnancy (February 2013). Pes planus was present at birth and she started to walk unaided at 16 months. In the family history, a first-degree cousin experienced early walking difficulties and hoarseness, and died at age of 9 years of respiratory failure, while still ambulant [Pedigree is reported in Fig. 1].

Our patient underwent orthopedic assessment at the age of 12 months and was then evaluated by a neurologist at 18 months. Neurological examination revealed bilateral facial weakness, reduced limb tone, muscle wasting prevalent in lower limbs, absence of tendon reflexes, proximal and distal weakness prevalent in lower limbs, hoarseness and inspiratory stridor. No palpable nerves were noted. Electrophysiological studies performed at 25 months revealed severe motor-sensory demyelinating neuropathy with secondary axonal loss [Ulnar nerve motor conduction velocity (CV) 27.1 m/s, CMAP 2 mV; Peroneal nerve motor CV 17.3 m/s, CMAP 0.4 mV; Median nerve sensory CV 19.4 m/s, SAP 2.8 µV; Sural nerve SAP not evocable].

In the following months neurological symptoms did not significantly evolve but the patient continued to present labored respiration with mild stridor. In January 2015 she underwent examination of the larynx by flexible videolaryngoscopy. The exam revealed left vocal cord palsy with right-sided hypomobility. Due to potential complication related to vocal cord paralysis, including respiratory insufficiency with possible aspiration and bronchopneumonia, the patient was considered a candidate for tracheostomy. However, considering the lack of progression of laryngeal symptoms in the following year and the presence of a normal polysomnography performed at 32 months [Oxygen desaturation events index (n°/h): 0.8. Minimum SatO_2_% value: 90, Mean SatO_2_% value: 94], surgical intervention was not performed and a regular follow-up was scheduled. Her laryngeal motility disorder was monitored by flexible videolaryngoscopies performed at intervals of 3–4 months. A progressive respiratory deterioration was observed with constant stridor for every minimal effort, activation of accessory respiratory muscles and inspiratory chest retractions, restlessness and difficulties during feeding. At the age of 3 years and 4 months a complete bilateral laryngeal paralysis was observed, with vocal cords in adduction and a respiratory space of about 1 mm. The parents refused surgical treatment at that time. At the age of 3 years and 7 months, due to worsening of clinical symptoms after an upper airway infection with severe respiratory obstruction, the patient required treatment in the Intensive Care Unit. Before admission the child was still able to walk unassisted with bilateral foot-drop and in the previous weeks she had started to present occasional dysphagia for both liquid and solid food. Muscle strength measured by MRC was 3–4 proximally in upper and lower limbs, 1–2 in the anterior tibialis and peroneus muscles. First, we performed glottic enlargement by direct microlaryngoscopy as an attempt to avoid permanent tracheotomy. After deep sedation, the vocal cords widely opened suggesting that the activity of vocal cords adductor muscles was a possible co-factor of the severe glottic obstruction. After incision of the mucosal surface of the posterior right vocal cord by CO_2_ laser, we partially vaporized the vocal muscle. Then, we placed two endo-extralaryngeal sutures by Lichtenberger needle carrier [4] in order to achieve lateralization of the posterior third of the vocal cord and to obtain closure of the cordotomy wound. We obtained the widening of the glottic space and extubated the child, who showed progressive respiratory improvement. A week after first surgery, the patient underwent tracheotomy as stridor had fully recurred and severe respiratory effort was persisting. Videolaryngoscopy showed the recurrence of glottic obstruction due to active adduction of the arytenoids during the whole respiratory cycle. After tracheotomy the child showed a general health improvement and resumed oral feeding.

### 2.2 Nerve biopsy and molecular findings

Sequencing analysis was initially performed in the *GDAP1* gene, responsible for autosomal recessive CMT4A and frequently associated with vocal cord paresis and stridor. As no mutations were detected, sural nerve biopsy was carried out at 20 months of age. Morphological analysis revealed reduced fiber number, several fibers with reduced myelin thickness, rare onion bulbs and the presence of abundant myelin outfoldings (Fig. 1), which are a hallmark of CMT4B neuropathy. Given the early onset of the neuropathy, *MTMR2* was first analyzed by performing Sanger sequencing analysis and a novel nonsense mutation in exon 6c.484 C>T; p.Arg162* was found. Consistent with an autosomal recessive inheritance, this mutation was present in homozygosity in the proband whereas unaffected parents were both heterozygous carriers of the mutation. This variant was not reported in the SNP database (dbSNP build 132, http://www.ncbi.nlm.nih.gov/projects/SNP) or in 1000 Genomes (www.1000genomes.org). The premature truncation of the MTMR2 protein at codon 162 leads to the deletion of part of the RID domain, the entire PTP-like phosphatase domain and the CC (coiled-coil) C-terminal motif [5], [6]. Moreover, mutations in the *MTMR13* gene responsible for CMT4B2, a severe early onset demyelinating neuropathy also characterized by myelin outfoldings in the nerve, were excluded in the proband as well.

## 3 Discussion

ARCMTs are usually associated with more severe phenotypes as compared to dominant forms [7]. Along with motor and sensory defects, they usually present earlier onset and more marked distal limb deformities, sometimes associated with major spinal abnormalities and other complications. Among CMT-associated complications, vocal cord paralysis is still an underestimated finding. Vocal cord paralysis has been reported as an early, severe and frequent symptom of GDAP1-associated neuropathy [CMT4A], often followed by diaphragmatic dysfunction [8], [9], [10], [11], [12], [13], and in both AD and AR forms of CMT2A (mutations in the *MFN2* gene) [14], [15]. Few cases are reported in axonal dominant CMT2C (mutations in the transient receptor potential cation channel, subfamily V, member 4; *TRPV4*) [16], [17], [18] and as a later involvement in a patient harboring a dominant heterozygous mutation of early growth response 2 (*EGR2*) gene [19]. Rarely, it has been associated with other variants and polymorphisms [20], [21], [22], [23], [24].

An overview of all previously reported cases with early onset in life is provided in Table 1.

Regarding CMT4B1, vocal cord paralysis was reported in one Italian patient, one English patient, and three Algerian siblings by Sabatelli [25], Tyson and Houlden [26], [27] and Nouioua [28], respectively. All of the patients suffered from early-onset (1–2 years-old) severe demyelinating neuropathy associated with respiratory difficulties. The Algerian siblings presented prominent chest deformities possibly secondary to breathing impairment. In the latter cases sequencing analysis revealed mutations located in exon 4 (c.G308A; p.Gly103Glu and c.331dupA; p.Arg111LysfsX24, respectively). Notably, laryngeal involvement was present in early stages of the disease in all cases.

In our patient, vocal cord paralysis presented as an early symptom, as the onset was between 1 and 2 years of age. The vocal cord disease manifested as a hoarse voice and stridor, associated with the use of accessory inspiratory muscles. An asymmetric involvement of vocal cords was initially noticed with a more severe paralysis of the left cord. This finding was present in previous reports [8], [20] and it has been interpreted as a sign of length-dependent degeneration of the neuropathy. However, initial paralysis of the right vocal cord is reported as well and usually a progression toward bilateral deficit is observed. Most of the genes involved in vocal cord paralysis encode for proteins playing a role also in neurons. We may speculate that vocal cord paralysis results from neuronal dysfunction in the bulbar nucleus of the recurrent nerve, rather than a consequence of a length-dependent neuropathy. This might also explain why only few CMT subtypes develop this complication and why no clear correlation with the severity of neuropathy is observed.

To our knowledge, no progression to respiratory failure was seen in previously reported cases. The patient reported by Tyson and colleagues, when aged 16, began to develop frequent chest infections and nocturnal hypoventilation. He was started on a nighttime BIPAP facial mask ventilator with resolution of symptoms. Nevertheless, as shown by our proposita, progressive laryngeal involvement may cause life-threatening airway obstruction, aspiration and pneumonia. Decreased life expectancy and premature death, which was previously described in other forms of CMT [5], [8], was observed also in our patient's first cousin, who was likely to harbor the same gene mutation (see genetic pedigree) and died of sudden death due to upper airway involvement at the age of 9, while he was still able to walk unaided.

In conclusion, we suggest that the finding of bilateral laryngeal paralysis or hypomotility due to unknown causes should prompt a warning for a neuromuscular disorder, notably a hereditary neuropathy, and help the physician to target molecular investigations. Particularly, we feel that laryngeal involvement might be a relevant and initial finding inearly-onset CMT4B1 neuropathy and thus all the patients with positive molecular genetics for *MTMR2* should be promptly investigated for laryngeal function. Periodic videolaryngoscopic follow-up is mandatory in patients with a CMT subtype associated with vocal cord paralysis in order to detect possible vocal cord hypomotility and to quantify the degree of airway obstruction. It is known that a young child can tolerate without symptoms a laryngotracheal obstruction up to 70% of the lumen, and that a slowly progressing obstruction may manifest with moderate stridor up to 80% or more [29]. Nevertheless, in such a clinical condition even a mild upper airway infection might have lethal consequences. Severe chest retractions, failure to thrive, refusal of food and no interest in playing might all be the signs of a critical condition which might rapidly precipitate [29]. Parents of the child should be made aware of their child condition in order to seek for hospital assistance as soon as they notice worsening of respiratory distress. In the described case, a first attempt to achieve a wider glottic space was performed by a vocal cord laterofixation procedure; this is a conservative mean of widening the posterior glottis respecting both phonatory and sphincteric functions of the larynx. Nevertheless, this procedure did not work in our patient, possibly due to the neuropathy as pathogenic mechanism of the vocal cord paralysis. Moreover, this case showed the role of active contraction of vocal cord adductor muscles as a cause of laryngeal obstruction in CMT patients. Tracheotomy appears then to be a necessary procedure to relieve the respiratory distress in patients affected by bilateral laryngeal paralysis in CMT.

## Figures and Tables

**Table 1:** Clinical reports of CMT cases associated with laryngeal involvement in the first 2 decades.

Reference	Gender/number of patients (age)	Country of origin	Age of walking (months)	Age of onset	Age of VF involvement	Cranial nerve involvement	Other peculiar features	Loss of ambulation (age)	Gene	CMT type, mode of inheritance
Present case	F/1 (3y)	Pakistan	16	12–18m	18m	Bilateral facial paresis, mild dysphagia	Pes planus	Still walking	MTMR2	CMT4B1, AR
Sabatelli et al., 1994 Luigetti et al., 2013	F/1 (28y)	Italy	13	14–15m	15m	Dysphagia, weakness of masticatory muscles, facial paresis	NA	22y	MTMR2	CMT4B1, AR
Tyson et al., 1997	M/1 (16y)	England	13	13–36m	36m	Difficulty with phonation, chewing, swallowing	Diaphragm paresis	16y	MTMR2	CMT4B1, AR
Nouioua et al., 2011	M/3 (8-16y)	Algeria	NA	1–2y	Stridor present at onset	Facial paresis	Chest deformities +++ Breathing difficulties. Pes equinovarus, claw hands	Still walking	MTMR2	CMT4B1, AR
Dyck et al., 1994	F/2 (26-47y)	NA	NA	6–8m	6-8m	Congenital III cranial nerve palsy	Short stature, diaphragm paresis	NA	TRPV4	CMT2C, AD
Chen et al., 2010	F/3 (44-73y); M/1 (29y)	Non-Hispanic white American	NA	1–10y	1-10y	NA	Short stature, diaphragm paresis	NA	TRPV4	CMT2C, AD
Echaniz-Laguna et al., 2014	M/10; F/7	France, Italy, Greece	NA	Childhood	NA	Hearing loss	Kyphoscoliosis, hyperCKemia, scapular winging, respiratory insufficiency, skeletal dysplasia, arthrogryposis, postural tremor	Still walking	TRPV4	CMT2C; CSMAA; dHMN ; SPSMA; AD – de novo -incomplete penetrance
Borkoel et al., 2003	F/1 (34y); M/1 (64y)	Japan; Costa Rica	15; 36	2y; 6m	NA	Facial paresis; dysphagia	NA	NA	GDAP1	CMT4A, AR
Sevilla et al., 2003	M/8; F/1 (33-57y)	France	12–18	Birth–2y	late	NA	NA	9y; 12y; 30y	GDAP1	CMT4A, AR
Azzedine et al., 2003	M/1 (30y)	Morocco	Normal	3y	20y	NA	Paralysis of one hemi diaphragm	15y	GDAP1	CMT4A, AR
Stojkovic et al., 2004	F/2 (32-33y)	France	20	<2 y	early in life	NA	Diaphragm paresis	25y	GDAP1	CMT4A, AR
Sevilla et al, 2008	M/6; F/3 (16-49y)	Spain	12–18	<1y–8y	NA	Bilateral facial weakness (n° 1); dysphagia (n° 1)	Diaphragm paresis	9- 38y (median 16)	GDAP1	CMT4A, AR
Moroni et al., 2009	M/1 (16y); F/1 (11y)	Italy	12–18	2y	13y	NA	NA	12y; still walking	GDAP1	CMT4A, AR
McEntagart et al., 2001 [30]	M/13; F/8	Wales	NA	2nd decade	2nd decade	NA	Distal muscular atrophy	NA	Dynactin	dHMN-VII, AD
Polke et al., 2011	F/1 (39y)	Italy	NA	3y	NA	Severe facial weakness, hearing loss, visual loss	Kyphoscoliosis, respiratory muscles weakness	Wheelchair (age not known)	MFN2	CMT2, AR
Bombelli et al., 2014	6	NA	NA	Early onset	NA	NA	Severe disability (5/6)	NA	MFN2	CMT2, AD
Pehlivan et al., 2015 [31]	M/1 († 33 m)	Turkey	Not acquired	<6m	<6m	NA	Lateral knee contractures, hammer toes, pes equinus and pes cavus	NA	TRIM2	AR

Abbreviations: AD, autosomal dominant; AR, autosomal recessive; CMT, Charcot–Marie–Tooth; CSMAA, congenital spinal muscular atrophy and arthrogryposis; dHMN, distal hereditary motor neuropathy; F, female; GDAP1, ganglioside-induced differentiation-associated protein 1; m, months; M, male; MFN2, mitofusin-2; MTMR2, myotubularin related protein 2; NA: data not available; SPSMA, scapula-peroneal spinal muscular atrophy; TRIM2, tripartite motif containing 2; TRPV4, transient receptor potential cation channel subfamily V member 4; VF, vocal fold; y, years; †, died.
